# Measuring misophonia in youth: A psychometric evaluation of child and parent measures

**DOI:** 10.1016/j.jad.2023.05.093

**Published:** 2023-05-30

**Authors:** Matti Cervin, Andrew G. Guzick, Jane Clinger, Eleanor E.A. Smith, Isabel A. Draper, Wayne K. Goodman, Marijn Lijffijt, Nicholas Murphy, Catherine E. Rast, Sophie C. Schneider, Eric A. Storch

**Affiliations:** a Department of Clinical Sciences Lund, Lund University, Sweden; b Department of Psychiatry and Behavioral Sciences, Baylor College of Medicine, Houston, TX, United States

**Keywords:** Misophonia, Children, Adolescents, Measures, Questionnaires, Psychometric, MAQ, A-MISO-S

## Abstract

**Background:**

Misophonia is characterized by intense emotional reactions to specific sounds or visual stimuli and typically onsets during childhood. An obstacle for research and clinical practice is that no comprehensively evaluated measures for pediatric misophonia exist.

**Methods:**

In a sample of 102 youth meeting the proposed diagnostic criteria of misophonia, we evaluated the child and parent-proxy versions of the self-reported Misophonia Assessment Questionnaire (MAQ; assessing broad aspects of misophonia) and the child version of the Amsterdam Misophonia Scale (A-MISO-S; assessing misophonia severity). Confirmatory and exploratory factor analysis were used to examine factor structures of the measures. Further, child-parent agreement on the MAQ and associations between both measures and misophonia-related impairment, quality of life, and misophonia-related school interference were examined to evaluate aspects of convergent validity.

**Results:**

For both youth- and parent-ratings, four MAQ factors emerged: pessimism, distress, interference, and non-recognition. A-MISO-S showed a unidimensional structure, but the item ‘effort to resist’ did not load significantly onto the unidimensional factor. Good child-parent agreement on the MAQ scales were found and both MAQ and A-MISO-S were moderately to strongly associated with misophonia-related impairment, quality of life (inverse association), and misophonia-related school interference.

**Limitations:**

MAQ and A-MISO-S assess sensitivity to auditory but not visual stimuli, the sample size was modest, and repeated assessments were not conducted.

**Conclusions:**

The combination of MAQ and A-MISO-S shows promise as a multidimensional assessment approach for pediatric misophonia. Future evaluations should include known-groups validity, screening performance, and sensitivity to change in symptom severity.

## 1. Introduction

Misophonia is an increasingly recognized condition characterized by intense emotional responses to specific sounds or visual stimuli (Swedo et al., 2022). Common misophonia triggers include sounds generated by other people, especially eating and breathing sounds, and repetitive mechanical sounds (e.g., tapping or clicking). In misophonia, these sounds evoke overwhelming experiences of anger, irritation, or panic (Guzick et al., 2023; Jager et al., 2020). (Guzick et al., 2023; Jager et al., 2020). Misophonia is related to but distinct from other conditions characterized by sound intolerance, for example, hyperacusis and phonophobia. Hyperacusis is a condition where an individual experiences marked physical discomfort at sound levels that are tolerable for most, while phonophobia refers to a persistent and unwarranted fear of specific sounds (Henry et al., 2022). In contrast, misophonia is not primarily a loudness tolerance problem, includes a broader range of triggers than just sounds (e.g., associated visual triggers), and elicits a wider range of emotional responses (often revolving around anger) (Jager et al., 2020).

The etiology of misophonia is unknown and there is no consensus about whether it is best considered an audiological, neurological, or psychiatric/psychological condition. Clinically, a multidisciplinary approach is recommended including otolaryngological, audiological, and psychological elements, and treatable medical causes should be ruled out; however, there are currently no agreement on specific assessment protocols to use (Henry et al., 2022; Palumbo et al., 2018). Misophonia typically begins during childhood (Jager et al., 2020; Rouw and Erfanian, 2018; Vitoratou et al., 2021) and pediatric misophonia is associated with significant psychiatric morbidity, functional impairment, and poor well-being (Guzick et al., 2023; Rinaldi et al., 2022). To date, there has been minimal validation of any misophonia measures for youth (Rinaldi et al., 2022). This is an important limitation as adequate assessment is a prerequisite for progress in the understanding of misophonia in children and adolescents.

For adults, a number of measures to assess misophonia have been developed and validated, most prominently the Duke Misophonia Scale (DMQ) (Rosenthal et al., 2021), the Selective Sound Sensitivity Syndrome Scale (S-Five) (Vitoratou et al., 2021), the Sussex Misophonia Scale (SMS; pre-print manuscript) (Rinaldi et al., 2021), the MisoQuest (Siepsiak et al., 2020), the Berlin Misophonia Scale-Revised (BMS-R) (Remmert et al., 2022), the Misophonia Response Scale (Dibb et al., 2021), and the Misophonia Questionnaire (Wu et al., 2014). Many of these measures were developed with multiple phases of validation including an initial large list of potential items with expert and stakeholder input and item reduction/retention decisions using item response theory and factor analysis; such methods were used for the DMQ (Rosenthal et al., 2021), the S-Five (Vitoratou et al., 2021), the BMQ (Remmert et al., 2022), and the Misophonia Response Scale (Dibb et al., 2021). Others, including the SMS and the MisoQuest, were conducted based on thorough literature reviews and proposed diagnostic criteria (Rinaldi et al., 2022; Siepsiak et al., 2020).

To our knowledge, only one misophonia measure has been evaluated in youth. A minimally adapted version of the SMS for adolescents (SMS-A) showed preliminary convergent and discriminant validity in a random sample of 10–14 year-olds (*n* = 142) and correlated moderately and significantly with measures of obsessive-compulsive symptoms, anxiety, and inversely with wellbeing, and did not show a significant correlation with creative self-concept (Rinaldi et al., 2021).

Because the SMS-A has only recently been validated and has not been tested in clinical samples or with a wider age range, existing case studies and case series of youth have relied on other, unvalidated measures. The most common measure is the Amsterdam Misophonia Scale (A-MISO-S, https://misophoniatreatment.com/wp-content/uploads/2014/06/A-MISO-S.pdf) (Schröder et al., 2013), which is based on the recommended symptom severity measure of obsessive-compulsive disorder, the Yale-Brown Obsessive-Compulsive Scale (Y-BOCS) (Goodman et al., 1989). A-MISO-S includes six items which, in line with Y-BOCS, assess time captured by symptoms, distress, interference, effort to resist symptoms, control over symptoms, and avoidance. In the only study that has evaluated the A-MISO-S in a younger age group, Naylor et al. (2021) found, in a sample of 336 undergraduate student (18–24 years), that the measure had a unidimensional factor structure, explaining 45 % of the variance in item responses, and adequate internal consistency, *α* = 0.81.

Another popular measure in case studies of youth with misophonia is the Misophonia Assessment Questionnaire (MAQ). This measure was developed by clinicians and assesses several aspects of misophonia-related experiences and functioning (Johnson and Dozier, https://misophoniatreatment.com/wp-content/uploads/2016/02/Binder_all_forms.pdf). Items on the MAQ address how misophonia might affect emotional functioning (e.g., “My sound issues have recently made me feel angry,” “My sound issues currently make me unhappy”), social functioning (“My sound issues currently make me feel isolated,” “My sound issues have recently affected my ability to be with other people”), as well as beliefs about the origin and prognosis of having sound sensitivity (e.g., “My sound issues do not seem to have a known cause,” “I feel that my sound issues will only get worse with time”). Although this measure has been used to draw preliminary conclusions about treatment outcomes in youth with misophonia via case series (Dozier, 2015; Lewin et al., 2021), psychometric validations have not been pursued.

Considering the lack of validated measures for misophonia in youth, the primary aim of this study is to psychometrically evaluate child- and parent-reported versions of the MAQ and a child-reported version of the A-MISO-S. Importantly, the measures will be evaluated in a sample of youth meeting the suggested diagnostic criteria for misophonia. Factor structures, internal consistency, child-parent agreement, and associations with misophonia-related impairment (clinician-rated), quality of life (child-rated), and misophonia-related school interference (parent-rated) will be examined. Given the lack of prior evaluations, the study will be conducted in an exploratory manner with the goal to provide empirical guidance about the measurement of misophonia in youth.

## 2. Methods

### 2.1. Participants and procedure

The study included 102 children and adolescents who met criteria for misophonia according to the Misophonia Assessment Interview (Lewin et al., 2021). The mean age of the sample was 13.69 years (SD = 2.50) and the mean age of misophonia onset was 8.8 years. A majority of participants identified as female (68 %), 28 % as male, one participant as a transgender male (1 %), one participant as a transgender female (1 %), and two participants as “other” (2 %). A vast majority of the sample identified as White (91 %). The sample has been described in detail elsewhere (Guzick et al., 2023).

Participants were recruited using flyers, website postings, Trialfacts, advertisement in extracurricular organizations and schools, and through Facebook groups for parents of children with misophonia. All participants expressing an interest to be included in the study were screened using a telephone interview conducted to determine whether the participant was likely to meet inclusion criteria which were (1) being 8–17 years old, (2) having a parent who was willing to participate, (3) fluency in English, and (4) clinically significant symptoms of misophonia. After screening, all participants underwent an online assessment that included the Misophonia Assessment Interview (MAI) (Lewin et al., 2021) and the Mini International Neuropsychiatric Interview for Children and Adolescents (MINI-KID) (Sheehan et al., 2010). All assessors were trained in the study instruments and had at least a bachelor's degree in psychology. During the assessment interview, participants completed online versions of study measures. The Institutional Review Board at Baylor College of Medicine approved the study and all participants provided written informed consent.

### 2.2. Measures

#### 2.2.1. Misophonia Assessment Questionnaire (MAQ)

As reviewed above, the MAQ is a 21-item measure of misophonia symptoms (Johnson and Dozier, https://misophoniatreatment.com/wp-content/uploads/2016/02/Binder_all_forms.pdf). Items are scored on 0–3 Likert scales. Rather than assessing misophonia severity per se, items on the MAQ assess potential negative impacts and experiences of misophonia (e.g., “My sound issues currently make me unhappy,” “My sound issues currently make me feel isolated,” “I feel that my sound issues will only get worse with time”). Both the child and parent-proxy versions were evaluated in this study. The parent version is identical to the child version but asks the parent to replace “my sound issues” with “my child's sound issues.” In a newer version of the MAQ (not available when the study was designed and conducted), “sound issues” has been replaced by “misophonia” to better capture the multi-sensory nature of misophonia (including visual triggers). The updated measure can be found here: https://misophoniatreatment.com/wp-content/uploads/2021/02/Misophonia_Assessment_Documents-9.pdf.

#### 2.2.2. Amsterdam Misophonia Scale (A-MISO-S)

The A-MISO-S is a 6-item (0–4 Likert scales) assessment tool of misophonia severity that has been used in both child and adult-focused research (Lewin et al., 2021; Schröder et al., 2013). The measure used items from Y-BOCS based on the hypothesis that misophonia may be an obsessive-compulsive spectrum disorder (Goodman et al., 1989; Lewin et al., 2021; Schröder et al., 2013). The six A-MISO-S items assess time taken by symptoms, distress, avoidance, control over misophonia-related thoughts, resistance to misophonia-related thoughts, and impairment. Both clinician-rated and self-rated versions have been used in prior research. In the present study, children rated the A-MISO-S in the presence of a trained assessor. A psychometric evaluation of a self-report version in undergraduate students showed a single-factor structure and adequate internal consistency, *α* = 0.81.

#### 2.2.3. Impairment rating of the Misophonia Assessment Interview

As part of the MAI (Lewin et al., 2021), all assessors rated the overall impairment stemming from misophonia using all available information. The impairment rating was conducted using a 0–8 item with a higher score indicating more impairment. This item was used as a convergent validator in the present study.

#### 2.2.4. Pediatric Quality of Life Enjoyment and Satisfaction Questionnaire (PQLESQ)

The PQLESQ is a 15-item self-report measure of quality of life for children and adolescents. It has shown strong internal consistency, test-retest reliability, and concurrent validity in youth (Endicott et al., 2006). The POLESQ score was used as a convergent validator in this study and showed good internal consistency, *a* = 0.90.

#### 2.2.5. Parent Responses to School Functioning Questionnaire (PRSFQ)

The PRSFQ is a parent-rated measure of school interference caused by their children's chronic pain (Garcia et al., 2017), and the original scale was worded for pain (e.g., “My child has the skills s/he needs to get though school despite pain”). For this study, items were modified to be misophonia-specific (e.g., “My child has the skills s/he needs to get though school despite misophonia”). PRSFQ has shown good internal consistency, test-retest reliability, criterion validity, and construct validity in a previous study about chronic pain in children (Garcia et al., 2017). In the present study, the PRSFQ was used as a convergent validator, and the scale showed good internal consistency, *a* = 0.83.

### 2.3. Statistical analysis

All statistical analyses were conducted in R Studio (version 2021.09.0) (R Core Team, 2020). First, we used confirmatory factor analysis (CFA) to test whether each of the measures could be considered to have a unidimensional factor structure. A unidimensional factor structure indicates that all items load onto a single latent factor, in this case, a factor representing overall misophonia severity. The CFAs were conducted using the R library *lavaan* (Rosseel, 2012). The items of all scales were ordinal and therefore the diagonally weighted least squares estimator was used. This estimator models non-normal responses as indicators of a normally distributed latent factors. Model/data fit was evaluated by inspecting four fit indices: Comparative Fit Index (CFI), Tucker-Lewis Index (TLI), Root Mean Square Error of Approximation (RMSEA), and Standardized Mean Square Residual (SRMR). An RMSEA below 0.06, an SRMR below 0.08 and CFI and TLI estimates >0.90 are indicative of acceptable model-data fit; CFI and TLI estimates above 0.95 are indicative of good model-data fit (Hu and Bender, 1999). We estimated scaled fit indices because of the ordinal nature of the data. Internal consistencies were estimated as part of the CFAs and Cronbach's alpha and McDonald's omega coefficients were used to examine internal consistency. Coefficients above 0.70 were considered adequate, coefficients above 0.80 were considered good, and coefficients above 0.90 were considered excellent.

If a unidimensional factor structure was not supported, we used exploratory factor analysis (EFA) to examine possible multidimensional factor structures. We estimated the correlation matrix of the items of each measure using Spearman's rho and computed the Kaiser-Meyer-Olkin (KMO) test values to examine whether the set of items and each individual item was suitable for EFA. KMO values indicate the proportion of variance in items that might be explained by latent factors; values above 0.80 are considered to indicate that EFA is well suited. The nScree function from the R library *nFactors* was used to determine the number of factors to retain during EFA. This function implements four methods to determine the appropriate number of factors: optimal coordinates, acceleration factor, parallel analysis, and Kaiser rule (Raiche et al., 2020). Factors were extracted using principal axis factoring and promax rotation. During extraction, factor loadings, RMSEA, and TLI were inspected to evaluate adequacy of fit. Associations between child- and parent-ratings of the MAQ and between both misophonia measures and clinician-rated misophonia-related impairment, child-rated quality of life, and parent-rated misophonia-related school impairment (i.e., convergent validators) were examined by estimating the correlation coefficients between the misophonia measures and the convergent validators.

## 3. Results

### 3.1. Factor structure

#### 3.1.1. MAQ

CFA with child-reported MAQ data indicated that a unidimensional factor structure had inadequate model/data fit, RMSEA = 0.11, CFI = 0.93, TLI = 0.92, SRMR = 0.11. All items loaded statistically significantly (*p*s < 0.001) onto the unidimensional misophonia factor with loadings ranging from 0.53 (item 5: *My sound issues do not seem to have a known cause*) to 0.90 (item 21: *I am worried that my whole life will be affected by sound issues*). The internal consistency of the unidimensional factor was excellent (*a* = 0.95, *ω* = 0.95) and the average item variance explained by the broad misophonia factor was 54 %.

When parent-rated data were used, the CFA with a unidimensional factor structure again indicated inadequate model/data fit, RMSEA = 0.11, CFI = 0.91, TLI = 0.90, SRMR = 0.11. All items loaded significantly onto the unidimensional misophonia factor with loadings ranging from 0.49 (item 5: *My child's sound issues do not seem to have a known cause*) to 0.83 (item 1: *My child's sound issues currently make him/her unhappy*). The internal consistency was excellent (*a* = 0.95, *ω* = 0.94) and the average item variance explained by the unidimensional factor was 52 %.

Because of the poor model/data fit of the unidimensional MAQ models, we proceeded with EFA. The overall KMO value for the child-rated data was 0.89 and the item-level KMO values ranged from 0.83 to 0.91. For the parent-rated data, the overall KMO value was 0.90 and the item-level values ranged from 0.87 to 0.93. Thus, KMO results indicated that both child and parent data were well-suited for EFA. The optimal coordinates, parallel analysis, and Kaiser rule methods indicated that a 4-factor solution was best in both child and parent data. Using child data, the four factors explained 59 % of the covariance among items and the correlations among the factors ranged from 0.52 to 0.62. The RMSEA was 0.06 and the TLI 0.93. Using parent-rated data, the four factors explained 58 % of the covariance among items and the correlations among the factors ranged from 0.29 to 0.64. The RMSEA was 0.04 and the TLI 0.97.

**Fig. 1.**
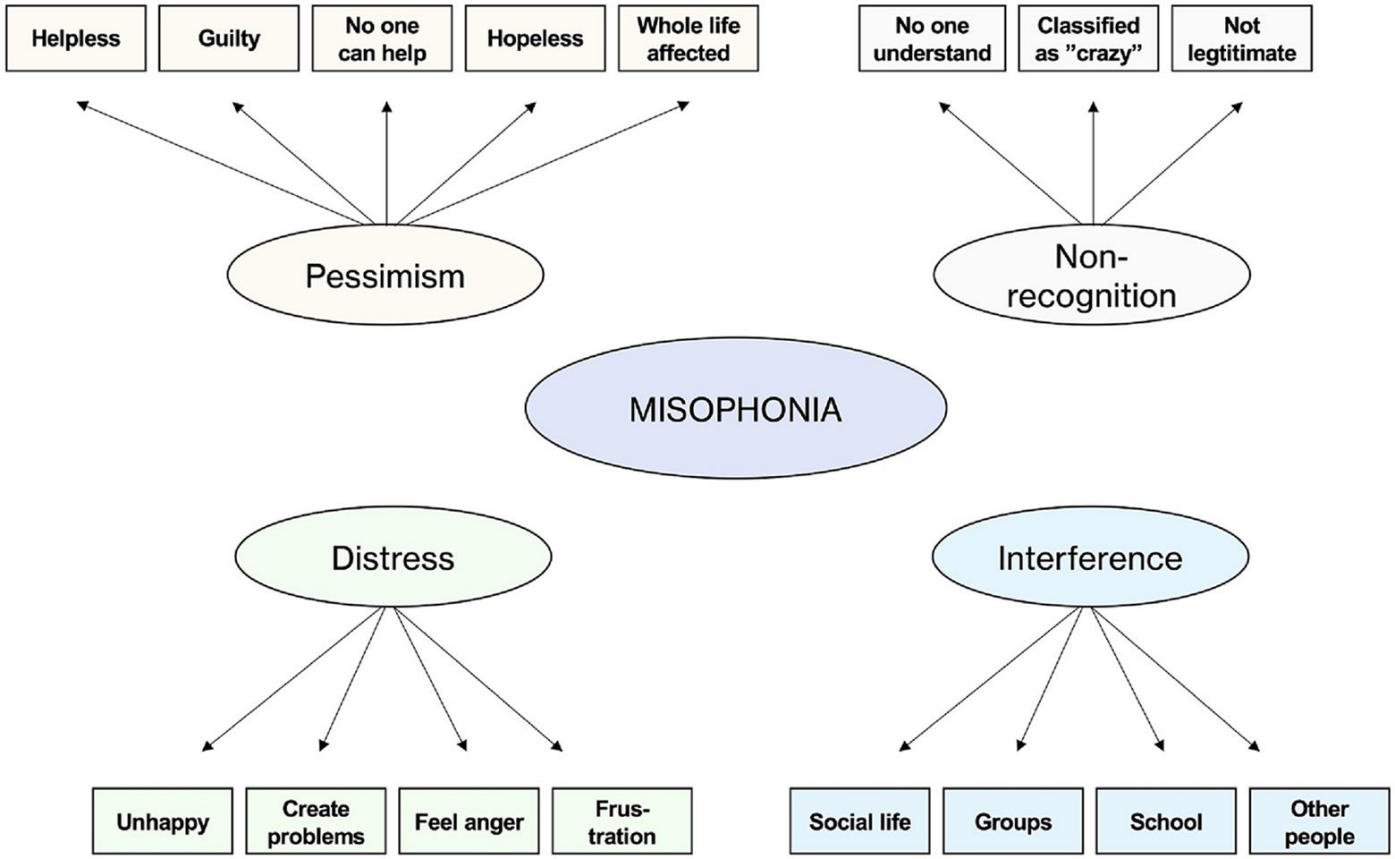
The EFA-derived four-factor structure of MAQ with 16 items showing identical loading in children and parents. *Notes*. EFA = Exploratory factor analysis. MAQ = Misophonia Assessment Questionnaire.

We extracted the 4 factors. The factor structures and item loadings for children and parents, respectively, are in Table 1. Similar structures emerged for child and parent data, with 16 of 21 items loading onto identical factors. We illustrate these four factors and their content in Fig. 1. Factor 1 was termed pessimism, factor 2 *current misophonia-related distress*, factor 3 *misophonia-related interference*, and factor 4 *non-recognition*. Items loading onto different factors across children and parents were item 5 (*My sound issues do not seem to have a known cause*, loading onto non-recognition for children and pessimism for parents), item 8 (*My sound issues currently make me feel isolated*, loading onto pessimism for children and interference for parents), item 12 (*My sound issues currently impact my entire life negatively*, not loading above 0.30 onto any factor for parents and onto pessimism for children), item 17 (*I feel that my sound issues will only get worse with time*, loading onto non-recognition for children and pessimism for parents), and item 18 (*My sound issues currently impact my family relationships*, loading onto interference for children and current distress for parents). Last, item 15 (*I feel that no one can help me with my sound issues*) loaded onto pessimism for children and parents but had a cross loading for children and loaded also onto non-recognition.

We tested the internal consistency of the four factors by including the 16 items that loaded onto the same factors in children and parents. Good to excellent internal consistency was found for all factors in children (*pessimism, a/ω*: 0.93/0.91, average item variance explained: 74 %; *non-recognition, a/ω*: 0.80/0.77, average item variance explained: 60 %; *interference*, alpha/omega = *a/ω*, average item variance explained: 69 %; current distress, *a/ω* = 0.89/0.85, average item variance explained: 70 %). Good to excellent internal consistency was also found for all factors in parents (*pessimism, a/ω* = 0.89/0.86, average item variance explained: 64 %; *non-recognition, a/ω* = 0.82/0.76, average item variance explained: 61 %; *interference, a/ω* = 0.87/0.83, average item variance explained: 64 %; current distress, *a/ω* = 0.90/0.86, average item variance explained: 72 %).

#### 3.1.2. A-MISO-S

The unidimensional factor structure of A-MISO-S showed poor model/data fit, RMSEA = 0.11, CFI = 0.95, TLI = 0.91, SRMR = 0.08. However, item 4 (*How much effort do you make to resist the [thoughts about the] misophonic triggers*?) did not load statistically significantly onto the unidimensional factor (standardized loading = −0.10, *p* = .93). When item 4 was removed, the model/data fit was adequate to good, RMSEA = 0.08, CFI = 0.99, TLI = 0.97, SRMR = 0.05. All items loaded significantly onto the broad factor with individual loadings from 0.60 (item 6, *Have you been avoiding doing anything, going any place, or being with anyone because of your misophonia?*) to 0.78 (item 2, *How much of your time is occupied by misophonic triggers?*). The scale had adequate internal consistency (*a/ω*: 0.81/0.76, average item variance explained: 47 %).

### 3.2. Convergent validity

Skewness and kurtosis values for all MAQ scales and A-MISO-S were between −1 and + 1 (except for child MAQ pessimism that had a kurtosis value of −1.19), indicating reasonable univariate normal distributions. Skewness and kurtosis for the three convergent validators were also within the −1 to +1 range. We therefore continued with Pearson product moment correlations for child-parent agreement and convergent validity.

#### 3.2.1. Child-parent agreement on MAQ

Correlations between the child and parent MAQ scales are presented in Table 2. All scales correlated statistically significantly and in the moderate to strong range, and the strongest correlations were found for corresponding scales (e.g., child-rated non-recognition with parent-rated non-recognition) except for child-rated current distress that had a slightly higher correlation with parent-rated pessimism (*r* = 0.54) than with parent-rated current distress (*r* = 0.52).

#### 3.2.2. Convergent validity between A-MISO-S and MAQ

The A-MISO-S correlated significantly (*p*s < 0.001) with the total score of both the child (*r* = 0.61) and parent (*r* = 0.62) versions of the MAQ. The A-MISO-S also correlated significantly (*p*s < 0.001) with all four subscales of the child MAQ (pessimism, *r* = 0.55; non-recognition, *r* = 0.36; interference, *r* = 0.47; current distress, *r* = 0.57) and parent MAQ (pessimism, *r* = 0.53; non-recognition, *r* = 0.36; interference, *r* = 0.48; current distress, *r* = 0.61).

#### 3.2.3. Convergent validity in relation to impairment, quality of life, and school interference

Correlations between the misophonia measures and clinician-rated misophonia impairment, child-rated quality of life, and parent-rated misophonia-related school interference are presented in Table 3. Clinician-rated impairment was significantly correlated with all misophonia measures with most correlations being in the moderate range and the strongest correlation emerging in relation to the child MAQ total score. Child-rated quality of life was also significantly and moderately correlated with all misophonia scales, with the strongest correlations emerging in relation to the child MAQ total score and the child MAQ pessimism subscale. Parent-rated misophonia-related school interference was significantly and moderately correlated with all misophonia measures except the child MAQ non-recognition scale; the strongest correlations emerged in relation to the child and parent MAQ interference subscales.

## 4. Discussion

Misophonia is increasingly recognized as an impairing condition and research with adults, including measurement development and evaluation, has burgeoned during the last years. While an increased focus on misophonia and its consequences is important, research with children and adolescents has lagged, which is unfortunate as the condition typically begins during childhood. A cornerstone in advancing the understanding of pediatric misophonia is adequate measurement of symptoms and their impact on everyday life and mental and social health. With the aim to inform research and support the assessment of misophonia in regular practice, we conducted a psychometric evaluation of two misophonia measures that have been used with youth but never evaluated: MAQ and A-MISO-S.

MAQ assesses negative effects of misophonia in everyday life as well as thoughts about the origins and prognosis of the sound difficulties. Our findings showed that the MAQ is best considered a multidimensional measure, with four identical scales emerging for the child and parent versions: pessimism, non-recognition, interference, and current distress. The four scales correlated in the moderate range with each other, indicating that they assess related but still distinct constructs. These findings provide the field with a four-dimensional model of misophonia that can be used in future research. A possible route is to examine whether difficulties in certain dimensions are more important to course and treatment response. The MAQ may also be used in regular practice to screen for different negative consequences of misophonia and possibly, although research is needed on known-groups validity, to identify youth suffering from this condition.

While very similar MAQ factors emerged for the child and parent versions, some differences for specific items were present. For example, the item that assesses current impact on family life loaded onto current distress for parents and interference for children. This indicates that some MAQ items take on slightly different meanings for children and parents. When examining child-parent agreement, it was clear that each MAQ subscale correlated most strongly with the corresponding child/parent subscale, providing evidence for convergent validity of the MAQ subscales outlined here. Although somewhat distinct, the four subscales correlated moderately to strongly with each other and there were only small differences in the size of these correlations. This suggests that the four scales can be considered indicators of a broad misophonia construct, which is well captured by the total MAQ score. Accordingly, the total score showed good convergent validity in relation to clinician-rated misophonia impairment, child-rated quality of life, and parent-rated school interference stemming from misophonia, as well as adequate internal consistency. We recommend future researchers to employ both the MAQ subscales and the MAQ total score in research on pediatric misophonia. However, it is important to evaluate whether the 4-factor structure presented here replicates in other youth samples.

The A-MISO-S is intended as a measure of overall misophonia severity and is based on a well-validated measure of OCD (Y-BOCS) (Goodman et al., 1989). The A-MISO-S assesses time, distress, interference, control, resistance, and avoidance. Our results support that a child-rated version of A-MISO-S shows promise as a unidimensional measure of overall severity of pediatric misophonia. Importantly, the measure was significantly associated with several other misophonia measures and with interview-rated misophonia-related impairment and school interference and inversely associated with quality of life. Unexpectedly, we found that item 4 (*How much effort do you make to resist the [thoughts about the] misophonic triggers*?) did not contribute with unique variance to the overall severity construct. When we examined item variance (not reported), we found that variance on this item was similar to the other A-MISO-S items. Thus, low variance could not explain the above result. It is worth noting that psychometric research on the Y-BOCS, upon which the A-MISO-S was based, has also found that the “resistance to obsessions” item has a subpar contribution to the full measure (Storch et al., 2010). This pattern appears to be even more pronounced when the item is applied to pediatric misophonia, and it may be that resistance to symptoms has more relevance to OCD than to misophonia. Further, when administering this item, it is likely that individuals have difficulty understanding what is meant by “resisting thoughts about misophonic triggers,” when a typical emotional response is often described as automatic and immediate rather than associated with very clearly identifiable cognitions. Particularly for younger children, it may be difficult to even conceptualize how they may resist thoughts about misophonic triggers. Further, in the present study, many participants had difficulties distinguishing this item from item five (how much control do you have over your thoughts about the sounds). We urge researchers using the A-MISO-S to carefully examine whether this and other items are useful indicators of overall misophonia severity and to continue to pursue developmentally sensitive adaptations of misophonia scales.

Strengths of this study include child and parent ratings from a well-defined and reasonably large sample of youth with interview-confirmed misophonia, but several limitations merit mentioning. First, although all participants were carefully assessed using the proposed criteria for misophonia, we did not conduct a multidisciplinary evaluation including assessments by an otolaryngologist and an audiologist, making it hard to completely rule out medical causes for the misophonia symptoms in each participant. Future studies should systematically examine whether and to what degree misophonia symptoms in children and adolescents can be explained by treatable medical causes. Second, although large in comparison with previous pediatric misophonia samples, the present sample did not allow for proper exploratory and confirmatory analyses, which would have been preferable. Third, almost all participants identified as White, limiting generalization of results. Fourth, no existing well-validated measures of pediatric misophonia exist, complicating convergent analyses. Fifth, measures were only completed at one timepoint and future research should examine test-retest reliability of the measures and their sensitivity to change in symptoms. A broader evaluation of the measures should also include a clinimetric perspective examining whether they capture all aspects of relevance to youth with misophonia and whether they are sensitive to change in disorder severity and can discriminate between clinically relevant subgroups (Fava et al., 2012). Last, the MAQ and A-MISO-S versions evaluated in the present study are limited by their emphasis on sounds and sound issues which excludes triggers of other sensory modalities (e.g., visual triggers). Further, the term sound issues can include difficulties related to hyperacusis, phonophobia, and/or tinnitus. Thus, individuals struggling with multiple sound-related conditions may therefore report their overall sound difficulties, rather than their misophonia-specific difficulties. Future research should carefully define misophonia and adjust assessment tools to capture the multi-sensory nature of the condition. Of note, an updated version of MAQ, which respects the multi-sensory nature of misophonia, has been developed and is freely available (see Methods section).

The present study provides empirical guidance about the measurement of misophonia in children and adolescents. By using the combination of MAQ and A-MISO-S, researchers can assess different aspects of pediatric misophonia using a short test battery. The child and parent versions of MAQ show very similar factor structures and the subscales have good internal consistencies and adequate convergent validity. The A-MISO-S shows promise as a unidimensional measure of pediatric misophonia severity but the item assessing resistance to misophonia triggers should possibly be excluded. Future research of these measures should include a broader definition of misophonia and evaluate the measures in larger samples with repeated assessments and in relation to clinically important factors (e.g., screening, treatment response) as well as in relation to outcomes that matter to youth with misophonia and their families.

## Figures and Tables

**Table 1:** Item factor loadings for factor structures derived through exploratory factor analysis for child- and parent-rated Misophonia Assessment Questionnaire data. Only factor loadings above 0.30 are shown. The items that load onto the same factor in both children and parents are highlighted in bold.

	Pessimism		Current Distress		Interference		Non-Recognition	
Item Content	Factor 1 Child	Factor 1 Parent	Factor 2 Child	Factor 2 Parent	Factor 3 Child	Factor 3 Parent	Factor 4 Child	Factor 4 Parent
1. My sound issues currently make me unhappy			**0.699**	**0.689**				
2. My sound issues currently create problems for me			**0.633**	**0.756**				
3. My sound issues have recently made me feel angry			**0.901**	**0.844**				
4. I feel that no one understands my problems with certain sounds							**0.700**	**0.462**
5. My sound issues do not seem to have a known cause		0.485					0.352	
6. My sound issues currently make me feel helpless	**0.731**	**0.602**						
7. My sound issues currently interfere with my social life					**0.845**	**0.730**		
8. My sound issues currently make me feel isolated	0.457					0.600		
9. My sound issues have recently created problems for me in groups					**0.783**	**0.909**		
10. My sound issues negatively affect my work/school life					**0.765**	**0.675**		
11. My sound issues currently make me feel frustrated			**0.600**	**0.587**				
12. My sound issues currently impact my entire life negatively	0.534				0.340			
13. My sound issues have recently made me feel guilty	**0.865**	**0.834**						
14. My sound issues are classified as ‘crazy’							**0.766**	**0.551**
15. I feel that no one can help me with my sound issues	0.487	0.545					0.607	
16. My sound issues currently make me feel hopeless	**0.794**	**0.815**						
17. I feel that my sound issues will only get worse with time		0.535					0.422	
18. My sound issues currently impact my family relationships				0.571	0.386			
19. My sound issues have recently affected my ability to be with other people					**0.811**	**0.512**		
20. My sound issues have not been recognized as legitimate							**0.717**	**0.891**
21. I am worried that my whole life will be affected by sound issues	**0.379**	**0.716**			0.358			

**Table 2:** Correlations between child and parent MAQ scales. All correlations are statistically significant at the *p* < .001 level. Corresponding scales are highlighted with bold.

	Parent Pessimism	Parent Interference	Parent Non-Recognition	Parent Current Distress	Parent Total Score
Child Pessimism	**0.61**	0.43	0.44	0.41	0.61
Child Interference	0.34	**0.49**	0.26	0.35	0.44
Child Non-Recognition	0.43	0.33	**0.65**	0.39	0.55
Child Current Distress	0.54	0.41	0.48	**0.52**	0.61
Child Total Score	0.60	0.50	0.54	0.49	**0.67**

*Notes.* MAQ = Misophonia Assessment Questionnaire.

**Table 3:** Correlations between misophonia scales and convergent validators.

	Interview -rated Misophonia Impairment	Child-rated Quality of Life, PQLESQ	Parent-rated school functioning, PRSFQ
Child A-MISO-S	0.55***	−0.26**	0.44***
Child MAQ Total Score	0.65***	−0.57***	0.32**
Child MAQ Pessimism	0.58***	−0.54***	0.28**
Child MAQ Interference	0.53***	−0.45***	0.47***
Child MAQ Non-Recognition	0.37***	−0.31**	0.06
Child MAQ Current Distress	0.60***	−0.43***	0.28**
Parent MAQ Total Score	0.60***	−0.37***	0.46***
Parent MAQ Pessimism	0.51***	−0.34***	0.36**
Parent MAQ Interference	0.54***	−0.31**	0.51**
Parent MAQ Non-Recognition	0.40***	−0.30**	0.27**
Parent MAQ Current Distress	0.48***	−0.21*	0.46**

A-MISO-S = Amsterdam Misophonia Scale. MAQ = Misophonia Assessment Questionnaire. PQLESQ = Pediatric Quality of Life Enjoyment and Satisfaction Questionnaire. PRSFQ = Parent Responses to School Functioning Questionnaire.

* *p* < .05.

** *p* < .01.

*** *p* < .001.
